# Dr. Simon L. Elsner

**Published:** 1910-07

**Authors:** 


Dr. Simon L. Elsner, a well-known surgeon of Rochester, died suddenly June 5, 1910, of angina pectoris while driving his automobile. When he collapsed his automobile swerved and was stopped by the steps at a church entrance. He was dead before a policeman could reach him. He graduated at the College of Physicians and Surgeons, New York, in 1887, and was forty-three years old at his death.

